# Anatomical Distribution of Diarrhetic Shellfish Toxins (DSTs) in the Japanese Scallop *Patinopecten yessoensis* and Individual Variability in Scallops and *Mytilus edulis* Mussels: Statistical Considerations

**DOI:** 10.3390/toxins10100395

**Published:** 2018-09-27

**Authors:** Ryoji Matsushima, Hajime Uchida, Ryuichi Watanabe, Hiroshi Oikawa, Izumi Oogida, Yuki Kosaka, Makoto Kanamori, Tatsuro Akamine, Toshiyuki Suzuki

**Affiliations:** 1 National Research Institute of Fisheries Science, Japan Fisheries Research and Education Agency, Fukuura 2-12-4, Kanazawa-ku, Yokohama, Kanagawa 236-8648, Japan; matsur@affrc.go.jp (R.M.); huchida@affrc.go.jp (H.U.); rwatanabe@affrc.go.jp (R.W.); oikawah@affrc.go.jp (H.O.); akabe@affrc.go.jp (T.A.); 2 Aomori Prefectural Industrial Technology Research Center, Fisheries Research Institute, Hiranai, Higashitsugarugun, Aomori 039-3381, Japan; izumi_oogida@aomori-itc.or.jp (I.O.); yuuki_kosaka@aomori-itc.or.jp (Y.K.); 3 Hokkaido Research Organization, Fisheries Research Department, Hakodate Fisheries Research Institute, Benten-cho 20-5, Hakodate, Hokkaido 040-0051, Japan; Kanamori-makoto@hro.or.jp

**Keywords:** diarrhetic shellfish toxins, accumulation, dinophysistoxin, Japanese scallop, dinophysis, LC/MS/MS, statistical analysis

## Abstract

Diarrhetic shellfish toxins (DSTs) are a group of phycotoxins that include okadaic acid (OA)/dinophysistoxin (DTX) analogues. At present, detailed data on the distribution of DST is insufficient, and studies of the appropriate sample sizes are lacking. This study investigated the DST frequency distribution in scallops and mussels by liquid chromatography-tandem mass spectrometry (LC/MS/MS) and a resampling analysis of existing data was carried out. The DST population-interval and the necessary sample size were also estimated. DSTs are localized in the scallop digestive-gland, and the DST concentrations in scallops were water-depth-dependent. DST concentrations in scallops and mussels showed normal distributions, but mussels tended to contain more DSTs than scallops. In the statistical resampling analysis of the acquired data on scallops and mussels, especially that using the bootstrap method, sample size was difficult to estimate when the DST variation was large. Although the DST population-interval could be statistically estimated from the sample standard deviation of three samples, the sample size corresponded to the risk management level, and the use of 13 or more samples was preferable. The statistical methods used here to analyze individual contents and estimate population content-intervals could be applied in various situations and for shellfish toxins other than DSTs.

## 1. Introduction

Diarrhetic shellfish poisoning (DSP) is a severe gastrointestinal illness caused by the consumption of shellfish contaminated with diarrhetic shellfish toxins (DSTs) [1]. DSTs are a group of phycotoxins that include okadaic acid (OA) and dinophysistoxin (DTX) analogues [2,3]. OA, dinophysistoxin-1 (DTX1), and dinophysistoxin-2 (DTX2) are the most important DSTs because they cause severe diarrhea. These toxins have been shown to be potent protein-phosphatase inhibitors [4], a property that can cause inflammation of the intestinal tract and diarrhea [5], possibly leading to tumor promotion [6]. Okadaic acid analogues are metabolized to the esterified toxin in many bivalve species including Japanese scallops [7], and they are collectively called dinophysistoxin-3 (DTX3). In Japan, screening and quantification of DSTs are carried out on bivalves in accordance with the guidelines based on the official instrumental method [8] in production areas and markets. However, the Japanese guidelines do not provide detailed information on the distribution of DSTs between individual bivalves and have not established an appropriate sample size due to the lack of such data obtained by accurate analytical methods [9].

The Japanese scallop *Patinopecten yessoensis* (*Mizuhopecten yessoensis*), a major and important cultured species in Japan [10], has unique characteristics, including the metabolic transformation of lipophilic toxins [11]. In the present study, we analyzed the concentrations of DSTs in individuals of *P*. *yessoensis* and the mussel *Mytilus edulis*, and the validity of the size of sample were examined with statistical resampling analysis of the acquired DST data. Although some of our research has been presented in a previous work [12], more detailed data and novel results are provided in our present study. Furthermore, assuming conditions for investigating cultured scallops in the sea [10], estimation of the DST population-interval (interval of concentration of DST contained in population) were performed. Finally, based on our results, we consider and propose an adequate sample size.

## 2. Results

### 2.1. Concentrations and Distribution of DSTs

#### 2.1.1. Anatomical Compartmentalization of DST in Scallops

The compartmentalization of DSTs in scallops collected at Nonai Station, Mutsu Bay, Aomori prefecture was investigated. From 14 to 20 individual scallops, the digestive gland, gonad, mantle, gill, and adductor muscle were separately dissected. The pieces were then grouped together by the body part (Table 1).

**Figure 1:**
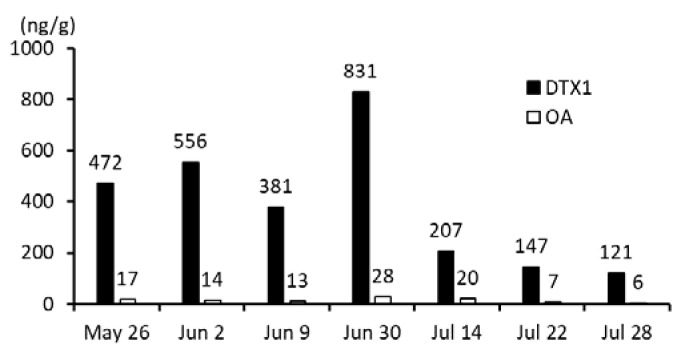
Concentrations of DTX1 and OA in the digestive glands of scallops. Fourteen to twenty individuals were combined into each sample set used for analysis. Black bars and white bars represent DTX1 and OA, respectively. The concentrations of toxins in the samples are shown on the vertical axis.

The dominant toxin in the scallops was DTX1, the highest concentration of which was found on 30 June, corresponding to about half of the regulation value of 0.16 mg/kg of whole meat (Figure 1).

**Figure 2:**
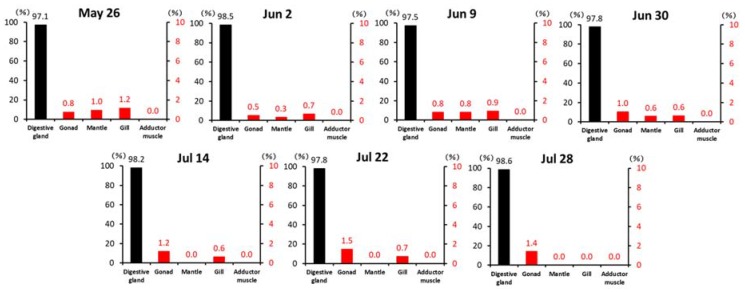
Percentage (%) of DTX1 in each scallop tissue.

The proportion of the DTX1 quantity corresponding to each tissue are shown in Figure 2.

#### 2.1.2. DST Analysis of 30 Individual Scallops and Mussels

**Figure 3:**
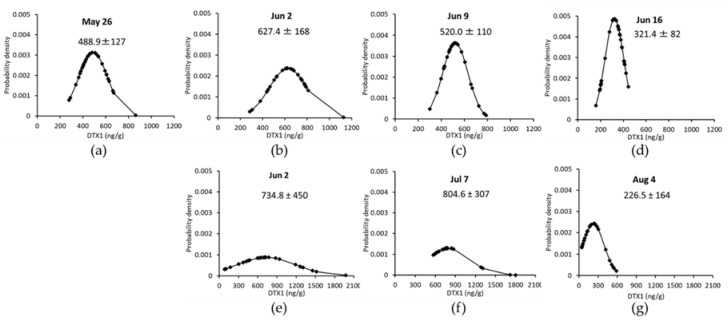
The 30 individual distributions, means ± *σ* of DTX1 in scallops and mussels. Panels (**a**–**d**) show the results for scallops and (**e**–**g**) show those for mussels.

The concentrations of DTX1 in the digestive glands of 30 scallops or mussels collected at the Nonai Station were quantified for each individual (Figure 3 and Table 2).

The concentration of OA was not described due to the overall low concentrations found in the individual samples. The mean values of DTX1 differed even for the same date for both mussels and scallops, and the DTX1 values of the mussels were higher than those of the scallops (Figure 3). The distributions of the scallops and mussels were close to the normal distributions.

#### 2.1.3. Analysis of DST Concentration in Scallop Samples from Different Water Depths

The DST concentrations of scallop digestive glands collected at different depths at Yakumo Station, in the western part of Funka Bay, Hokkaido prefecture, were investigated. Sampling information about the scallops is shown in Table 3.

**Figure 4:**
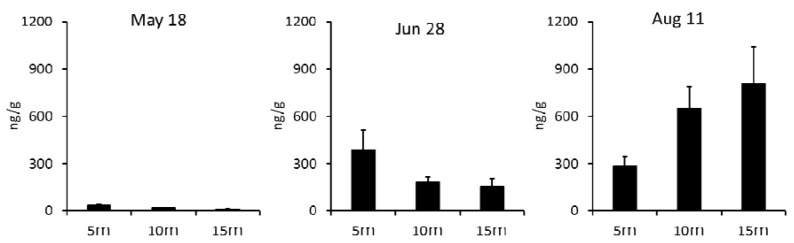
Vertical distribution of DTX1 and *σ* in scallop digestive glands.

The vertical gradient of DTX1 distribution (maximum at 5 m, minimum at 15 m) was reversed over the investigation period (Figure 4). Maximum levels of about half of the regulation value (corresponding to 0.16 mg/kg of whole meat) were found at 15 m on 11 August.

**Figure 5:**
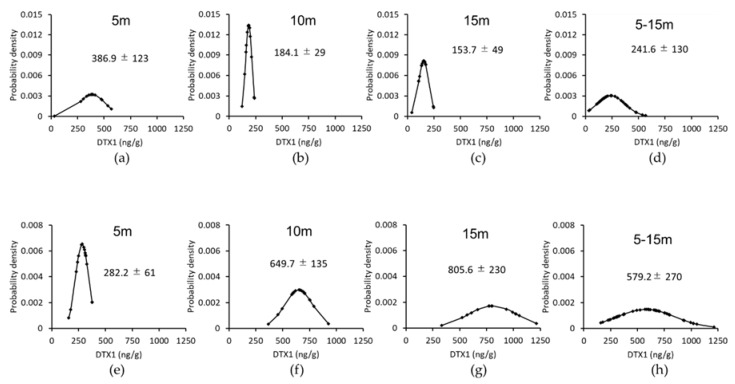
The distributions of scallop at each depth on 28 June and 11 August. The mean concentrations of DTX1 ± *σ* are shown for (**a**–**d**) 28 June and (**e**–**h**) 11 August.

The distributions of scallops on 28 June and 11 August were close to the normal distribution (Figure 5).

**Figure 6:**
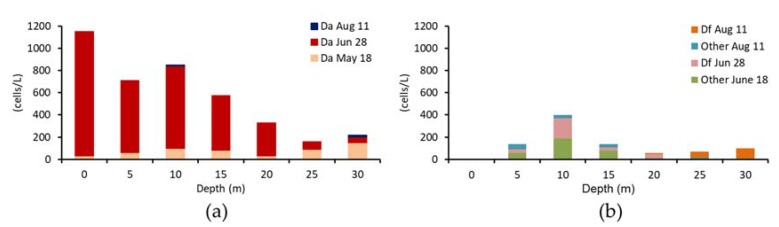
Vertical distributions of *Dinophysis species*. (**a**) *D*. *acuminata*; (**b**) *D*. *fortii* and other *Dinophysis species*.

Environmental conditions and vertical distribution of *Dinophysis* at Yakumo Station are represented in Table 4 and Figure 6, respectively.

*D*. *acuminata* was assumed to be main causative agent of the DST events. This species reached its maximal density on 28 June and declined on 11 August (Table 4). Other *Dinophysis* species were predominant from 10 m to the surface, and there was no clear relationship between the distribution of cells and reversal of the vertical distribution of DTX1 in scallops on 11 August (Figure 4 and Figure 6).

### 2.2. Statistical Analysis

#### 2.2.1. Statistical Resampling Analysis of DSTs in Scallops and Mussels

The number of individuals necessary to correctly reflect the DST contents of collected samples was estimated by resampling analysis. For this purpose, we used a data set collected for 30 individual scallops and 30 individual mussels at Aomori on 2 June (Figure 3). Both values were highly variable. The means of 5–25 samples were calculated with random sampling and allowing or not allowing (bootstrap method) replacement. The sample means were converted to percentages as compared with those of 30 samples (Table 5).

In the resampling analysis without replacement, using ≥8 and ≥17 scallops fell within ±20% and 10% of the means of 30 individuals, respectively, with a probability of 98%. In the case of mussels, means of ≥13 and ≥19 individuals fell within ±30% and 20% of the means of 30 individuals, respectively, and with 98% probability (Table 5, underlined numbers in the left half). In the bootstrap method, only 11 scallops fell within ±20% of the mean value of 30 individuals with a probability of 98% (Table 5, double-underlined numbers in the right half), whereas the number of mussels was not obtained from 25 individuals.

#### 2.2.2. Estimating the Mean Concentration of the Population (Cultured Scallops) When the Individual Concentration of a Sample Is Defined

In the former subsection, the ratio of the sample mean to the 30-individual population DST mean was analyzed using specific individual samples. Here, the mean DST concentration of the population (cultured scallops) was estimated using the sample mean concentration. Estimation of the standard normal distribution population mean with the confidence interval (CI, 95% = 100 (1 − *α*)%) is represented by the following equation [13]:

(1) $$\;\bar{X}-1.96\times \sqrt{\mathrm{Var}\left(\bar{X}\right)}\leq \hat{\mu }\leq \bar{X}+1.96\times \sqrt{\mathrm{Var}\left(\bar{X}\right)}\;$$

By the central limit theorem, variance Var(X¯)=σ2n** [13]**.

(2)  X¯−1.96×σ2n≤μ^≤X¯+1.96×σ2n 

The unknown-population standard deviation σ can be replaced with the sample standard deviation s calculated from the sample data. Moreover, 1.96 = *t*_0.05_(∞) and hence is generalized with $t_{\alpha }\left(\nu \right)$.

(3) $$\;\bar{X}-t_{\alpha }\left(\nu \right)\times s/\sqrt{n}\;\leq \hat{\mu }\leq \bar{X}+t_{\alpha }\left(\nu \right)\times s/\sqrt{n}\;$$

In Equation (3), $t_{\alpha }\left(\nu \right)\times s/\sqrt{n}\;$ (CI of the population mean concentration) was estimated when s was 200 at [*α* = 0.05] using 10 samples. In accordance with *n* and *α*, *t*_0.05_(9) = 2.2622 from the Student’s *t*-distribution in Table 6 was assigned in Equation (3).

(4) $$\;t_{\alpha }\left(\nu \right)\times s/\sqrt{n}=\;2.2622\;\times \;200/\surd 10\;=\;143.1\;$$

At *α* = 0.05 and 0.10, the interval estimations (±OA group ng/g) of μ with sample sizes of 3–20 and *s* of 100–650 were calculated and are presented in Table 7 and Table 8.

From Table 7 and Table 8, it is possible to estimate the confidence interval of the μ when *s* is 100–650 for 3–20 samples, when the individual concentration data of the sample are acquired.

From Equation (5), derived from Equation (3), the s value can be calculated to estimate the mean density of μ within interval ±160 ng/g (one-tenth of the digestive gland regulatory limit).

(5) $$\;t_{\alpha }\left(\nu \right)\times s/\sqrt{n}\;\leq 160\;$$

(6) $$\;s\leq 160/t_{\alpha }\left(\nu \right)\times \sqrt{n}.\;$$

In Equation (5), the ***s*** was estimated using *t*_0.05_(9) = 2.2622 from Student’s *t*-distribution table.

(7) $$\;s\leq 160/t_{\alpha }\left(\nu \right)\times \sqrt{n}=\;160/2.2622\;\times \;3.1622\;=\;223.7\;$$

**Figure 7:**
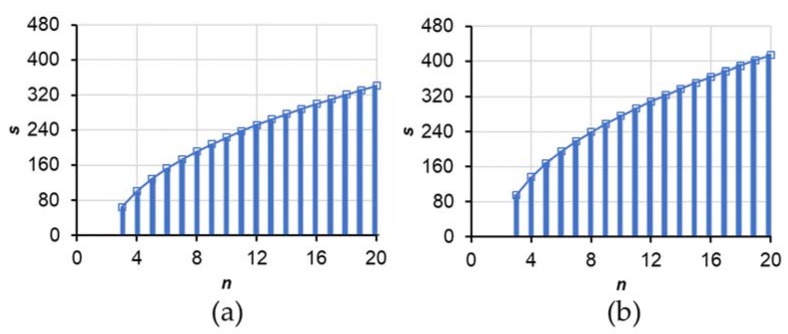
Estimation of *s* with the mean density of μ within interval ±160 ng/g at (**a**) 95% or (**b**) 90% confidence. The X axis *n* represents a sample size of 3–20.

At *α* = 0.05 and 0.10, the estimation of *s* with the mean density of *μ* within interval ±160 ng/g with sample sizes of 3–20 were calculated and are presented in Figure 7.

For an actual sample, when the calculated ***s*** is less than or equal to the graph value (striped zone of Figure 7), it is considered that the mean concentration of μ can be estimated with an interval of ±160 ng/g and 90% or 95% confidence.

#### 2.2.3. Adequacy of Sample Size Based on the *t*-Value and Confidence Interval

The values in Table 7 and Table 8 and in Figure 7 are derived by equations with a *t*-value as a coefficient. *n* is a natural number, and the statistical degrees of freedom (*ν*) is a linear function. On the other hand, the *t*-value is defined by Equation (6) [14,15], and the upper cumulative probability (1/2 *α*) of the *t* distribution is derived from Equation (7).

(8) $$\;f\left(t\right)=\;\frac{\Gamma \left(\frac{\nu +1}{2}\right)}{\sqrt{\nu \pi }\Gamma \left(\frac{\nu }{2}\right)}{\left(1+\frac{t^{2}}{\nu }\right)}^{-\frac{\nu +1}{2}}\;\left(\Gamma \;\mathrm{is}\;\mathrm{Gamma}\;\mathrm{function}\right)\;$$

(9) $$\;1/2\alpha =\overset{\infty }{\underset{x}{\int }}f\left(t,\;\upsilon \right)dt\;$$

**Figure 8:**
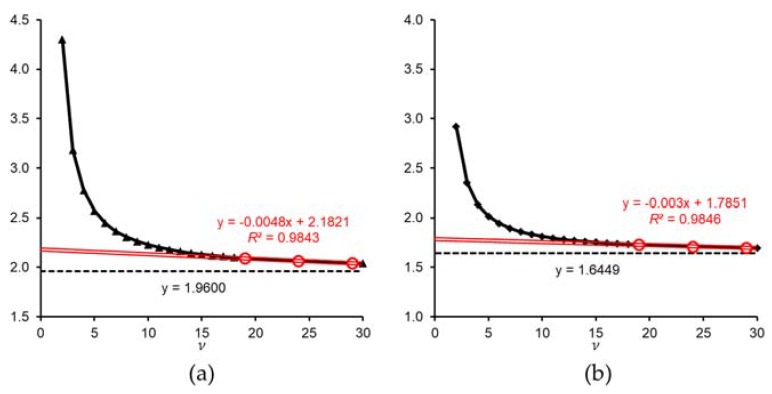
The *t*-value of *α* = 0.05 or 0.10 with *ν* = 2–30. (**a**) Triangles are *t*_0.05_(2–30); (**b**) diamonds are *t*_0.10_(2–30). The dotted lines show (**a**) y = 1.9600 and (**b**) y = 1.6449. The red circles represent the *t*-values of *n* = (20, 25, 30) at *α* = 0.05 or 0.10, and red double lines approximate the straight line of each *t*_0.05_(20, 25, 30) and *t*_0.10_(20, 25, 30). The red equations on the graph represent a linear approximation line of *t*-values.

In Equation (7), although the *t*-value (*x*) is determined by the degrees of freedom *ν* and significance level *α*, it is inversely correlated with the *ν* and *α*, and the *t*-value (*x*) gradually approaches infinite degrees of freedom at each significance level. Figure 8 represents the *t*-value of *α* = 0.10 or 0.05 with *ν* = 2–30. The *t*-value increases as the confidence value becomes greater, without intersection.

Ultimately the line is nearly straight with zero slope passing through the *t*-value at each infinity *ν* (*t*_0.10_(∞) = 1.6449 or *t*_0.05_(∞) = 1.9600 equal to the standard normal distribution at each confidence level). When *t* = 0.05 or 0.10, points of *n* = 20, 25, 30 and approximate straight lines are drawn on a *t*-value graph (Figure 8, red double lines). Although the coefficient of approximate straight lines varies depending on the desired value and confidence, the risk of the obtained estimation value is greatly reduced as the sample size increases and the *t*-value approaches each linear approximation.

Both approximate straight lines start to diverge from the *t*-value around *ν* = 13 (*n* = 14), and the *t*-value increases exponentially as *ν* decreases. From the samples of about 14 or more, the relation between the risk of the estimated value and the size of the sample is assumed to describe a linear function. Thus, it is desirable to use a sample size of at least 14 when estimating the mean concentration of the population. When the sample size is 13 or less, the risk of the estimate value increases exponentially as the sample size decreases.

**Figure 9:**
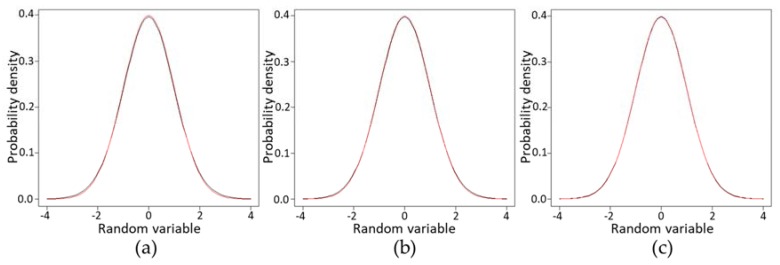
Overlay graphs of *f*(*t*, *ν* = 30, 50, and 100) red lines, and the standard normal distribution *f*(*t*, *ν* = ∞) black lines. (**a**) *f*(*t*, *ν* =30) and *f*(*t*, *ν* = ∞); (**b**)* f*(*t*, *ν* = 50) and *f*(*t*, *ν* = ∞); (**c**) *f*(*t*, *ν* = 100) and *f*(*t*, *ν* = ∞).

The graph of* f*(*t*, *ν* = 100) and the standard normal distribution *f*(*t*, *ν* = ∞) are almost identical (Figure 9c). The difference between *t*_0.10_(100) and* t*_0.10_(∞) or that between *t*_0.05_(100) and* t*_0.05_(∞) is 0.0153 or 0.024, respectively (Table 6). These levels are within a margin of error that does not matter practically. The ideal number of samples is 14 (*ν* = 13) or more, but considering the mathematical errors, 13 (*ν* = 12) or more samples is assumed to be a practical allowable range.

## 3. Discussion

Some *Dinophysis* species in Japan produces DTX1 as the dominant toxin [7,16]. In scallops, results showing that DSTs were detected exclusively in the digestive glands and that the adductor muscles were free of DSTs agree with the results of our previous feeding experiment study [11].

In our samples, the ratio of the digestive glands to the whole meat of the mussels was about 15% (data from three groups of 30 mussel samples). Some of the mussels collected on 2 June and 7 July exceeded the regulatory level, but none of the scallop samples exceeded the regulatory level. Even though the scallops and mussels were cultured in the same spot, the amounts of accumulated DSTs and their variabilities tended to be higher in mussels than in scallops. The mussels adhered to each other via the byssus, whereas the scallops were separated from each other in the net. Ecological factors and metabolism may be involved in the magnitude of DST variability in mussels and scallops. Concentration fluctuations and DST variation determined by water depth (Figure 4) seem to reflect the vertical distribution of *Dinophysis* in the water column and the individual metabolism of scallops. The peak of *Dinophysis* species cell density was assumed to have occurred in July, but unfortunately our data did not identify this trend.

Given a sufficient sample size, it is common practice to homogenize and prepare samples, and this makes sense from the viewpoint of equalizing the samples. Generally, according to the law of large numbers, when an appropriate sample size is collected, there is no problem in obtaining the mean value of the population even if the samples are combined. However, this theorem does not present the validity of the sample size, and in fact it is a problem that convergence of mean value requires plenty of samples. Hence, there is important question as to what the pooled sample size should be. It should also be noted that valuable information on the toxin concentrations in individual shellfish flesh is lost by homogenization and cannot be used to estimate risk.

In the current DST testing of scallops or mussels, it is not feasible to collect 30 individuals, so the number of individuals necessary to reflect the mean DST content of 30 samples collected were estimated in scallops and mussels by statistical resampling analysis using the actual values. Approximately 8 or more samples were considered adequate for scallops, as the variation in DTX1 levels was less than that of mussels. In the bootstrap analysis of more highly variable mussels, 25 samples were insufficient to fall within ±30% of mean value with a probability of 98%. The sample size required changes according to the desired degree of uncertainty level, and the calculation results are restricted to those specific already-known sample data.

As mentioned above, usually a sample size of 30 is difficult to obtain for scallops or mussels. Therefore, we used statistical methods to estimate the mean value of the general population under more practical conditions. Estimation of the population by statistical processing and estimation of the size of necessary samples are basic methods that are described in some textbooks [13,17]. However, no cases have been applied to the field of shellfish toxins. To estimate the mean value of the DST populations of shellfish samples, the samples must be distributed normally in order to apply the statistical parametric test equation. From the individual data group (Figure 3 and Figure 5), the DSTs of each sample basically have a one-peak distribution in which the histogram is almost symmetrical, and the mean and median values are almost matched. Thus, regardless of the concentration, when the variation was not extremely large, it was considered normally distributed. Moreover, it is necessary to take as random a sample as possible from the fact that the total of the depth distribution graph with 5–15 m takes a more normal distribution (Figure 5d,h).

According to the calculation results of Equations (3) and (5) (Table 7 and Figure 7), if there is individual information about the samples, the risk can be evaluated as a concrete figure for the mean concentration of the population. Even when the sample size is less than 5, the interval estimation against DST can be obtained from the value. This makes it possible to evaluate whether sampling is sufficient or not as well as the risk of estimating population concentration. Attention should be paid to the difference between *σ* and *s* in these statistical calculations. The statistical definition of *σ *is completely different from that of *s*, and *s* is required for estimating the value. Although in this report we did not deal directly with other shellfish toxins such as paralytic shellfish toxins (PST), the mean concentration of the population and the risk can be estimated and evaluated according to Equation (3) in the same manner.

As the sample size increases in *t*-value, the slope of the linear approximation decreases and the value becomes strict (Figure 8). Finally, it is a straight line with zero slope passing through the infinity *t*-value with the necessary reliability (ex, y = *t*_0.10_(∞) = 1.6449 or y = *t*_0.05_(∞) = 1.9600). The larger the sample size, the better, but in practice there are many cases where statistical ideals are not satisfied due to various restrictions. In the case of scallops or mussels, calculation of an approximate expression using an impractically large size of samples is irrational and not applicable to real-world conditions. Hence, the linear approximate expression at each reliability (*α* = 0.10 or 0.05) was calculated using samples of 20, 25, and 30 in this study (Figure 8). Samples of about 14 or more for scallops or mussels were derived as ideal sample sizes as the result of the estimation from Figure 8. Because the graph of *f*(*t*, *ν* = 100) and the standard normal distribution *f*(*t*, *ν* = ∞) are almost identical (Figure 9), 13 or more samples are considered to be a practical preferred range including mathematical errors.

On the other hand, in an investigation or analysis that can sample from 100 to 1000 individuals, another criterion corresponding to such sample size should be applied. A small sample size such as *n* = 3 and a high risk of *α* = 0.10 are presented in this report for research purposes. The desired minimum sample size is 13 or more, and it is necessary to carefully consider the risk corresponding to *α* = 0.10, meaning a rejection rate of 10%.

In conclusion, our study shows that DSTs in scallops and mussels are localized in the digestive gland and the DST concentrations have a normal distribution. Statistical analysis of the normal distribution data enables estimation of DST population-interval and shows that a sample size of 13 or more individuals is desirable. Simple evaluations and calculations using tables in this article can be applied in various situations. Although it is inevitable to combine samples at the time of the actual inspection of shellfish, in research, it is desirable to try to acquire as much important individual information as possible, to obtain more accurate values, and to evaluate the risks. Those results are used as an index for risk assessment and are expected to contribute to risk management in shellfish toxins.

## 4. Materials and Methods 

### 4.1. Plankton Monitoring

One liter of seawater was sampled from May to August 2016 using Van Dorn bottles at 5 m depth intervals from Yakumo Station (42°16.208′ N, 140°20.568′ E) in Uchiura Bay (Funka Bay), Hokkaido, Japan. Each 1 L sample was concentrated and resuspended to 10 mL by filtration through a 20-μm mesh plankton net sieve and fixed with 1.25% glutaraldehyde. To estimate the densities of *Dinophysis*, 1 mL of each sample was stained with 0.01% of fluorescent dye (Whitex BB, Sumitomo Chemical, Chuo-ku, Tokyo, Japan) and observed with an inverted epifluorescence microscope (IX71, Olympus, Shinjuku-ku, Tokyo, Japan) under UV light excitation. Vertical profiles of temperature and salinity were obtained from CTD (RINKO-Profiler ASTD102, JFE Advantech, Nishinomiya, Hyogo, Japan) casts. Water transparency was recorded using the Secchi disk (30 cm in diameter, RIGO CO. LTD., Bunkyo-ku, Tokyo, Japan).

### 4.2. Scallops and Mussels

Scallops and mussels, several individuals grown on each lantern net [10] at the same point, were collected from Nonai Station (40°52′ N, 140°07′ E, Depth = 32 m) in Mutsu Bay, Aomori Prefecture, Japan, in 2014. The Aomori prefecture is located at the northern end of Honshu Island. Other scallops with ear-hanging [10] were harvested near Yakumo Station (42°16.558′ N, 140°20.000′ E) of Uchiura Bay, Hokkaido, Japan, in 2016. Hokkaido is the northernmost prefecture of Japan.

### 4.3. Extraction of DSTs and Hydrolysis of Esterified DSTs

Each dissected tissue was homogenized with 9 volumes of methanol-distilled water (9:1, *v*/*v*), and the homogenates were centrifuged at 3000 rpm for 5 min [18]. Alkaline hydrolysis of the OA group was carried out according to the EU harmonized standard operating procedure for lipophilic marine biotoxins in molluscs by LC-MS/MS, ver. 5 [19]. For hydrolysis, 125 μL of 2.5 M NaOH solution was added to a 1 mL aliquot of a methanolic extract of each sample. The mixture was kept at 80 °C for 30 min and neutralized with 125 μL of 2.5 M HCl. The hydrolyzed samples were analyzed by LC/MS/MS without further purification.

### 4.4. Standard Toxins

The National Metrology Institute of Japan certified reference material of okadaic acid (OA) and dinophysistoxin-1 (DTX1) [20] were dissolved in HPLC-grade methanol to prepare the calibration standards.

### 4.5. LC/MS/MS Analysis of DSTs

OA and DTX1 in sample extracts were analyzed and quantified by LC/MS/MS as reported previously [16,18]. Triplicate analyses were carried out for each sample extract. Multiple reaction monitoring (MRM) LC/MS/MS analysis for toxins was carried out using [M − H]^−^ as target parent ions in Q1 and particular fragment ions of each toxin in Q3, with a dwell time of 100 ms for each analogue as follows. OA: *m/z* 803.5 > 255.3; DTX1: *m/z* 817.5 > 255.3. LoD (limit of detection) of OA and DTX1 < 0.01 mg/kg. The proportion of the DTX1 quantity corresponding to each tissue was calculated by multiplying the concentrations by the total tissue weight.

### 4.6. Statistical Analyses

Statistical analysis program R [21] with boot (https://CRAN.R-project.org/package=boot) and MASS (https://CRAN.R-project.org/package=MASS) packages were employed for resampling analysis and for graphing the standard normal distribution and *t*-distribution. The values repeatedly calculated 10,000 times with 5–25 random samplings from 30 scallops or mussels were converted to percentages as compared with the mean value of 30 scallops or mussels. This computation is a kind of bootstrap method that has been modified so as not to allow replacement. This provides an estimate of the mean distribution, and how the mean varies depending on the size of samples is presented. The bootstrap method is also applied in the same manner except to allow replacement. Since the resampling analysis without replacement picks up different random samples and the bootstrap method may pick up random samples, including the same samples, more stringent results can be obtained.

Population is parent population.

Sample size is the number of individuals within a group. Number of samples is the number of groups.

*N* = number of individuals in the population.
*n* = number of individuals in the sample.

The population mean is represented by the Greek letter *mu* (μ), and $\hat{\mu }$ represents the estimator.

The $x_{i}$ is an individual sample value, and the sample mean is represented by $\bar{x}$.

The population variance is denoted by $\sigma^{2}$.

(10) $$\;\sigma^{2}=\frac{1}{N}\overset{N}{\underset{i=1}{\sum }}{\left(x_{i}-\mu \right)}^{2}\;$$

The population standard deviation is denoted by σ.

(11) $$\;\sigma =\sqrt{\sigma^{2}}\;$$

The sample variance is denoted by $s^{2}$.

(12) $$\;s^{2}=\frac{1}{n-1}\overset{n}{\underset{i=1}{\sum }}{\left(X_{i}-\bar{X}\right)}^{2}\;$$

The sample standard deviation is denoted by s.

(13) $$\;s=\sqrt{s^{2}}.\;$$

Two-tailed significance level (*α*) = 0.05 or 0.10.
Confidence level = 1 − *α.*
Confidence interval (CI) = 100 (1 − *α*)%.
Statistical degrees of freedom = *n* − 1 = Greek letter *nu *(*ν*).
*t* is derived from Student’s *t*-distribution table using *n* − 1 and *α* (Table 6).

According to the central limit theorem, regardless of the distribution of the population, if the sample number is made sufficiently large, the error between the population mean and the sample mean follows a normal distribution.

According to the law of large numbers, if the sample size is made sufficiently large, the sample mean converges to the population mean.

## Figures and Tables

**Table 1:** Sampling information and total weight (g) of each scallop tissue.

2014	26 May	2 June	9 June	30 June	14 July	22 July	28 July
Number of Individuals	16	18	17	18	15	20	14
Digestive gland	72.90	72.87	72.97	70.80	60.80	75.16	58.56
Gonad	42.39	39.99	40.79	44.22	48.05	54.46	39.83
Mantle	155.01	151.37	163.48	175.80	163.66	213.96	169.30
Gill	95.31	93.43	106.28	92.20	87.08	116.42	85.49
Adductor muscle	301.69	315.19	311.42	355.14	318.80	434.35	340.26

The concentration of OA and DTX1 in each part was quantified by LC/MS/MS after hydrolysis.

**Table 2:** Sampling information and mean weight (g) of the digestive glands of 30 scallops or mussels. The mean values of 30 samples ± population standard deviation (*σ*).

2014	26 May	2 June	9 June	16 June	7 July	8 August
Scallop (Digestive gland/Whole meat %)	3.87 ± 1.07 (10.14%)	3.62 ± 0.81 (10.56%)	3.70 ± 0.98 (8.78%)	3.81 ± 0.72 (9.18%)	-	-
Mussel (Digestive gland/Whole meat %)	-	1.69 ± 0.45 (14.72%)	-	-	1.40 ± 0.46 (14.89%)	1.51 ± 0.45 (12.90%)

**Table 3:** Sampling information and mean weight (g) of scallop digestive glands. The mean value of digestive glands ± *σ* at each depth.

2016	18 May	28 June	11 August
Number of Individuals	10	15	15
5 m	6.45 ± 1.24	4.77 ± 0.82	1.40 ± 0.36
10 m	6.16 ± 0.91	5.18 ± 1.33	1.38 ± 0.26
15 m	5.30 ± 0.45	4.22 ± 0.75	1.46 ± 0.42

**Table 4:** Data on environmental conditions and densities of DST producing species at Yakumo Station.

Transparency (m)	Date (2016)	Depth (m)	Water Temperature (°C)	Salinity (psu)	*D*. *fortii *(Cells/L)	*D*. *acuminate *(Cells/L)	Other *Dinophysis *(Cells/L)	
	18 May	0	11.2	30.84	0	30	0	
		5	9.7	31.98	0	60	0	
5.0		10	9.2	32.03	0	100	0	
		15	8.1	32.32	0	80	0	
		20	7.8	32.59	0	30	0	
		25	7.4	32.67	0	90	0	
		30	7.3	32.70	0	150	0	
	28 June	0	16.6	29.58	0	1120	0	
		5	14.8	31.15	30	650	60	Dt60
4.0		10	13.7	31.94	180	740	190	Dn150, Dt40
		15	13.4	32.09	30	490	80	Dn60, Dr20
		20	13.0	32.18	40	300	10	Dr10
		25	12.8	32.22	0	70	10	Dn10
		30	12.3	32.33	0	50	10	Dn10
	11 August	0	22.6	31.14	0	0	0	
		5	20.7	31.67	0	0	50	Dt50
10.5		10	16.9	32.37	0	10	30	Dt30
		15	12.7	32.64	0	0	30	Dt20, Dr10
		20	10.8	32.95	10	0	0	
		25	8.6	32.95	50	0	10	Dt10
		30	7.9	33.02	90	20	0	

*Dinophysis tripos *(Dt)*; Dinophysis norvegica *(Dn)*; Dinophysis rotundata* (Dr).

**Table 5:** Resampling analysis of scallops and mussels without replacement and with the bootstrap method. The left half of the table is a resampling analysis without replacement, while the right half shows the data using the bootstrap method. The n columns represent 5–25 samples. 1 to 99 represent percentiles. Percentage: each percentile columns represents the mean value of a data set for each mean of the 30 individuals. >±*30*; <±30; <±**20**; <±**10**.

	Scallop				Mussel					Scallop				Mussel			
n	1	5	95	99	1	5	95	99	n	1	5	95	99	1	5	95	99
**5**	75.6	**82.1**	**119.0**	126.3	*49.5*	*60.4*	*144.4*	*163.7*	**5**	72.5	**80.7**	120.7	*130.3*	*44.3*	*58.2*	*149.8*	*172.0*
**6**	77.7	**84.0**	**117.0**	123.7	*52.0*	*63.9*	*139.4*	*155.8*	**6**	75.6	**82.3**	**118.8**	127.2	*48.8*	*61.3*	*144.6*	*164.2*
**7**	80.0	**85.4**	**114.9**	120.4	*56.2*	*66.9*	*134.8*	*148.8*	**7**	76.6	**83.5**	**116.8**	124.4	*51.3*	*63.8*	*139.8*	*157.2*
**8**	**81.1**	**86.5**	**113.9**	**119.0**	*58.5*	*69.5*	*132.6*	*144.4*	**8**	78.4	**84.7**	**115.7**	122.9	*54.9*	*66.5*	*137.3*	*154.3*
**9**	**82.9**	**87.6**	**112.9**	**117.6**	*61.9*	71.8	129.8	*140.3*	**9**	79.2	**85.5**	**114.3**	121.1	*56.5*	*67.8*	*133.9*	*150.0*
**10**	**84.4**	**88.5**	**111.6**	**116.0**	*64.1*	73.7	126.9	*138.6*	**10**	**80.7**	**86.3**	**114.0**	120.1	*59.4*	*69.8*	*132.6*	*147.9*
**11**	**85.5**	**89.3**	**111.0**	**114.7**	*67.0*	75.9	124.8	*135.4*	**11**	**81.4**	**86.8**	**113.3**	**119.1**	*60.6*	70.4	*131.4*	*145.4*
**12**	**86.1**	**89.8**	**110.4**	**114.1**	*69.4*	77.4	123.7	*132.1*	**12**	**82.4**	**87.4**	**112.9**	**118.8**	*62.5*	72.0	*130.7*	*144.1*
**13**	**87.4**	**90.7**	**109.4**	**112.9**	70.6	78.5	121.6	129.7	**13**	**82.9**	**87.9**	**112.2**	**117.6**	*63.5*	73.0	128.8	*140.9*
**14**	**87.8**	**91.2**	**108.6**	**112.1**	72.5	79.7	**119.9**	127.7	**14**	**83.6**	**88.5**	**112.1**	**117.4**	*65.3*	74.2	128.7	*140.8*
**15**	**88.7**	**91.7**	**108.0**	**111.1**	74.2	**81.2**	**118.9**	126.3	**15**	**84.5**	**88.9**	**111.6**	**116.3**	*65.6*	75.1	126.8	*137.8*
**16**	**89.7**	**92.5**	**107.6**	**110.3**	75.0	**82.2**	**117.5**	124.2	**16**	**84.6**	**89.2**	**111.0**	**115.6**	*66.8*	75.9	126.0	*136.9*
**17**	**90.0**	**92.8**	**107.1**	**109.7**	77.6	**83.7**	**116.5**	122.4	**17**	**85.1**	**89.5**	**110.7**	**115.1**	*67.2*	76.6	124.9	*136.2*
**18**	**90.8**	**93.2**	**106.5**	**109.1**	78.5	**84.8**	**115.1**	120.4	**18**	**85.8**	**89.8**	**110.6**	**115.2**	*68.9*	77.2	124.3	*135.3*
**19**	**91.4**	**93.6**	**106.2**	**108.4**	**80.0**	**85.6**	**113.8**	**119.2**	**19**	**86.0**	**90.0**	**110.2**	**114.7**	*69.9*	77.5	123.9	*134.4*
**20**	**92.0**	**94.0**	**105.8**	**107.8**	**81.1**	**86.7**	**112.9**	**117.3**	**20**	**85.8**	**90.2**	**109.9**	**114.0**	*69.5*	78.0	123.2	*133.6*
**21**	**92.6**	**94.5**	**105.2**	**107.2**	**82.2**	**87.4**	**111.9**	**116.0**	**21**	**86.8**	**90.6**	**109.6**	**113.9**	70.5	78.8	122.6	*132.3*
**22**	**93.1**	**94.9**	**104.9**	**106.6**	**83.7**	**88.4**	**110.8**	**114.5**	**22**	**87.1**	**90.8**	**109.4**	**113.6**	71.0	79.2	122.0	*132.7*
**23**	**93.5**	**95.2**	**104.5**	**106.3**	**84.7**	**89.2**	**110.1**	**113.5**	**23**	**87.2**	**91.0**	**109.3**	**113.2**	72.0	79.8	121.6	*131.1*
**24**	**94.1**	**95.7**	**104.0**	**105.5**	**86.1**	**90.2**	**108.8**	**111.7**	**24**	**87.4**	**91.0**	**109.2**	**113.0**	72.3	79.9	121.3	*130.5*
**25**	**94.6**	**96.1**	**103.6**	**105.0**	**87.3**	**91.1**	**107.9**	**110.3**	**25**	**88.0**	**91.3**	**109.1**	**113.1**	73.3	**80.5**	121.1	*130.4*

**Table 6:** Student’s *t* distribution.

*ν*	Two-Tailed Probability	
	0.10	0.05
2	2.9200	4.3027
3	2.3534	3.1824
4	2.1318	2.7764
5	2.0150	2.5706
6	1.9432	2.4469
7	1.8946	2.3646
8	1.8595	2.3060
9	1.8331	2.2622
10	1.8125	2.2281
11	1.7959	2.2010
12	1.7823	2.1788
13	1.7709	2.1604
14	1.7613	2.1448
15	1.7531	2.1314
16	1.7459	2.1199
17	1.7396	2.1098
18	1.7341	2.1009
19	1.7291	2.0930
20	1.7247	2.0860
21	1.7207	2.0796
22	1.7171	2.0739
23	1.7139	2.0687
24	1.7109	2.0639
25	1.7081	2.0595
26	1.7056	2.0555
27	1.7033	2.0518
28	1.7011	2.0484
29	1.6991	2.0452
30	1.6973	2.0423
50	1.6759	2.0086
100	1.6602	1.9840
∞	1.6449	1.9600

The two-tailed probability is 0.10 or 0.05.

**Table 7:** Interval estimation (±OA group ng/g digestive gland) of μ in *α* = 0.05. The *n* columns represent sample size 3–20 samples. Rows from 100 to 650 represent the *s* values.

*n*	*t* (*α* = 0.05)	Sample Standard Deviation (*s*)											
		100	150	200	250	300	350	400	450	500	550	600	650
**3**	4.3027	248.4	372.6	496.8	621.0	745.2	869.5	993.7	1117.9	1242.1	1366.3	1490.5	1614.7
**4**	3.1825	159.1	238.7	318.3	397.8	477.4	556.9	636.5	716.1	795.6	875.2	954.8	1034.3
**5**	2.7764	124.2	186.2	248.3	310.4	372.5	434.6	496.7	558.7	620.8	682.9	745.0	807.1
**6**	2.5706	104.9	157.4	209.9	262.4	314.8	367.3	419.8	472.2	524.7	577.2	629.7	682.1
**7**	2.4469	92.5	138.7	185.0	231.2	277.5	323.7	369.9	416.2	462.4	508.7	554.9	601.1
**8**	2.3646	83.6	125.4	167.2	209.0	250.8	292.6	334.4	376.2	418.0	459.8	501.6	543.4
**9**	2.3060	76.9	115.3	153.7	192.2	230.6	269.0	307.5	345.9	384.3	422.8	461.2	499.6
**10**	2.2622	71.5	107.3	143.1	178.8	214.6	250.4	286.1	321.9	357.7	393.5	429.2	465.0
**11**	2.2281	67.2	100.8	134.4	167.9	201.5	235.1	268.7	302.3	335.9	369.5	403.1	436.7
**12**	2.2010	63.5	95.3	127.1	158.8	190.6	222.4	254.1	285.9	317.7	349.5	381.2	413.0
**13**	2.1788	60.4	90.6	120.9	151.1	181.3	211.5	241.7	271.9	302.1	332.4	362.6	392.8
**14**	2.1604	57.7	86.6	115.5	144.3	173.2	202.1	231.0	259.8	288.7	317.6	346.4	375.3
**15**	2.1448	55.4	83.1	110.8	138.4	166.1	193.8	221.5	249.2	276.9	304.6	332.3	360.0
**16**	2.1315	53.3	79.9	106.6	133.2	159.9	186.5	213.2	239.8	266.4	293.1	319.7	346.4
**17**	2.1199	51.4	77.1	102.8	128.5	154.2	180.0	205.7	231.4	257.1	282.8	308.5	334.2
**18**	2.1098	49.7	74.6	99.5	124.3	149.2	174.0	198.9	223.8	248.6	273.5	298.4	323.2
**19**	2.1009	48.2	72.3	96.4	120.5	144.6	168.7	192.8	216.9	241.0	265.1	289.2	313.3
**20**	2.0930	46.8	70.2	93.6	117.0	140.4	163.8	187.2	210.6	234.0	257.4	280.8	304.2

**Table 8:** Interval estimation (±OA group ng/g digestive gland) of μ in *α* = 0.10. The *n* columns represent sample size 3–20. Rows 100 to 650 represent the *s* values.

*n*	*t* (*α* = 0.10)	Sample Standard Deviation (*s*)											
		100	150	200	250	300	350	400	450	500	550	600	650
**3**	2.9200	168.6	252.9	337.2	421.5	505.8	590.1	674.3	758.6	842.9	927.2	1011.5	1095.8
**4**	2.3534	117.7	176.5	235.3	294.2	353.0	411.8	470.7	529.5	588.4	647.2	706.0	764.9
**5**	2.1318	95.3	143.0	190.7	238.3	286.0	333.7	381.3	429.0	476.7	524.4	572.0	619.7
**6**	2.0150	82.3	123.4	164.5	205.7	246.8	287.9	329.0	370.2	411.3	452.4	493.6	534.7
**7**	1.9432	73.4	110.2	146.9	183.6	220.3	257.1	293.8	330.5	367.2	404.0	440.7	477.4
**8**	1.8946	67.0	100.5	134.0	167.5	201.0	234.4	267.9	301.4	334.9	368.4	401.9	435.4
**9**	1.8595	62.0	93.0	124.0	155.0	186.0	216.9	247.9	278.9	309.9	340.9	371.9	402.9
**10**	1.8331	58.0	87.0	115.9	144.9	173.9	202.9	231.9	260.9	289.8	318.8	347.8	376.8
**11**	1.8125	54.6	82.0	109.3	136.6	163.9	191.3	218.6	245.9	273.2	300.6	327.9	355.2
**12**	1.7959	51.8	77.8	103.7	129.6	155.5	181.5	207.4	233.3	259.2	285.1	311.1	337.0
**13**	1.7823	49.4	74.1	98.9	123.6	148.3	173.0	197.7	222.4	247.2	271.9	296.6	321.3
**14**	1.7709	47.3	71.0	94.7	118.3	142.0	165.7	189.3	213.0	236.6	260.3	284.0	307.6
**15**	1.7613	45.5	68.2	91.0	113.7	136.4	159.2	181.9	204.6	227.4	250.1	272.9	295.6
**16**	1.7530	43.8	65.7	87.7	109.6	131.5	153.4	175.3	197.2	219.1	241.0	263.0	284.9
**17**	1.7459	42.3	63.5	84.7	105.9	127.0	148.2	169.4	190.5	211.7	232.9	254.1	275.2
**18**	1.7396	41.0	61.5	82.0	102.5	123.0	143.5	164.0	184.5	205.0	225.5	246.0	266.5
**19**	1.7341	39.8	59.7	79.6	99.5	119.3	139.2	159.1	179.0	198.9	218.8	238.7	258.6
**20**	1.7291	38.7	58.0	77.3	96.7	116.0	135.3	154.7	174.0	193.3	212.7	232.0	251.3

## References

[1] Yasumoto T., Oshima Y., Yamaguchi M. (1978). Occurrence of a new type of shellfish poisoning in the Tohoku. Bull. Jpn. Soc. Sci. Fish..

[2] Yasumoto T., Murata M., Oshima Y., Sano M., Matsumoto G.K., Clardy J. (1985). Diarrhetic shellfish toxins. Tetrahedron.

[3] Yasumoto T., Murata M. (1993). Marine toxins. Chem. Rev..

[4] Bialojan C., Takagi A. (1988). Inhibitory effect of a marine-sponge toxin, okadaic acid, on protein phosphatases. Specificity and kinetics. Biochem. J..

[5] Terao K., Ito E., Yanagi T., Yasumoto T. (1986). Histopathological studies on experimental marine toxin poisoning. I. Ultrastructural changes in the small intestine and liver of suckling mice induced by dinophysistoxin-1 and pectenotoxin-1. Toxicon.

[6] Fujiki H., Suganuma M., Suguri H., Yoshizawa S., Takagi K., Uda N., Wakamatsu K., Yamada K., Murata M., Yasumoto T. (1988). Diarrhetic shellfish toxin, dinophysistoxin-1, is a potent tumor promoter on mouse skin. Jpn. J. Cancer Res..

[7] Suzuki T., Ota H., Yamasaki M. (1999). Direct evidence of transformation of dinophysistoxin-1 to 7-*O*-acyl-dinophysistoxin-1 (dinophysistoxin-3) in the scallop *Patinopecten yessoensis*. Toxicon.

[8] Guidelines for Risk Management of Shellfish Toxins in Bivalves. http://www.maff.go.jp/j/syouan/tikusui/gyokai/g_kenko/busitu/pdf/150306_kaidoku_guide.pdf.

[9] Standard for Liver and Raw Bivalve Molluscs (CODEX STAN 292-2008). http://www.fao.org/fao-who-codexalimentarius/sh-proxy/es/?lnk=1&url=https%253A%252F%252Fworkspace.fao.org%252Fsites%252Fcodex%252FStandards%252FCODEX%2BSTAN%2B292-2008%252FCXS_292e_2015.pdf.

[10] Sandra E.S., Parson J.G. (2016). Scallops: Biology, Ecology, Aquaculture, and Fisheries.

[11] Matsushima R., Uchida H., Nagai S., Watanabe R., Kamio M., Nagai H., Kaneniwa M., Suzuki T. (2015). Assimilation, accumulation and metabolism of dinophysistoxins (DTXs) and pectenotoxins (PTXs) in the Japanese scallop *Patinopecten yessoensis*. Toxins.

[12] Matsushima R., Uchida H., Watanabe R., Oikawa H., Kosaka Y., Tanabe T., Suzuki T. (2018). Distribution of Diarrhetic Shellfish Toxins in Mussels, Scallops, and Ascidian. Food Saf..

[13] Lemeshow S., Hosmer D.W., Klar J., Lwango S.K. (1990). World Health Organization. Adequacy of Sample Size in Health Studies.

[14] Student (1908). The probable error of a mean. Biometrika.

[15] Fisher R.A. (1925). Applications of “tudent’s” distribution. Metron.

[16] Suzuki T., Miyazono A., Baba K., Sugawara R., Kamiyama T. (2009). LC–MS/MS analysis of okadaic acid analogues and other lipophilic toxins in single-cell isolates of several *Dinophysis* species collected in Hokkaido, Japan. Harmful Algae.

[17] (May 1993). Sampling Plans for Aflatoxin Analysis in Peanuts and Corn.

[18] Suzuki T., Jin T., Shirota Y., Mitsuya T., Okumura Y., Kamiyama T. (2005). Quantification of lipophilic toxins associated with diarrhetic shellfish poisoning in Japanese bivalves by liquid chromatography-mass spectrometry and comparison with mouse bioassay. Fish. Sci..

[19] EU-Harmonised Standard Operating Procedure for Determination of Lipophilic Marine Biotoxins in Molluscs by LCMS/MS Version 5. http://www.aecosan.msssi.gob.es/AECOSAN/docs/documentos/laboratorios/LNRBM/ARCHIVO2EU-Harmonised-SOP-LIPO-LCMSMS_Version5.pdf.

[20] NMIJ CRM Catalog 2016–2017, National Institute of Advanced Industrial Science and Technology (AIST), National Metrology Institute of Japan (NMIJ). https://www.nmij.jp/english/service/C/CRM_Catalog_(JE)160901.pdf.

[21] R: A Language and Environment for Statistical Computing. https://www.R-project.org/.

